# Identification of the Secreted Proteins Originated from Primary Human Hepatocytes and HepG2 Cells

**DOI:** 10.3390/nu11081795

**Published:** 2019-08-03

**Authors:** Andras Franko, Sonja Hartwig, Jörg Kotzka, Marc Ruoß, Andreas K. Nüssler, Alfred Königsrainer, Hans-Ulrich Häring, Stefan Lehr, Andreas Peter

**Affiliations:** 1 Department for Diagnostic Laboratory Medicine, Institute for Clinical Chemistry and Pathobiochemistry, University Hospital Tübingen, 72076 Tübingen, Germany; 2 Institute for Diabetes Research and Metabolic Diseases of the Helmholtz Centre Munich, University of Tübingen, 72076 Tübingen, Germany; 3 German Center for Diabetes Research (DZD e.V.), 85764 Neuherberg, Germany; 4 Institute for Clinical Biochemistry and Pathobiochemistry of DDZ, University of Düsseldorf, 40225 Düsseldorf, Germany; 5 Department of Traumatology, BG Trauma Clinic, Siegfried Weller Institute for Trauma Research, Eberhard Karls Universität Tübingen, 72076 Tübingen, Germany; 6 Department of General, Visceral and Transplant Surgery, University Hospital Tübingen, 72076 Tübingen, Germany; 7 Department of Internal Medicine IV, Division of Endocrinology, Diabetology, Nephrology, University Hospital Tübingen, 72076 Tübingen, Germany

**Keywords:** mass spectrometry, proteomics, primary human hepatocytes, HepG2 cells, hepatokines

## Abstract

The liver plays a pivotal role in whole-body carbohydrate, lipid, and protein metabolism. One of the key regulators of glucose and lipid metabolism are hepatokines, which are found among the liver secreted proteins, defined as liver secretome. To elucidate the composition of the human liver secretome and identify hepatokines in primary human hepatocytes (PHH), we conducted comprehensive protein profiling on conditioned medium (CM) of PHH. Secretome profiling using liquid chromatography-electrospray ionization tandem mass spectrometry (LC-MS/MS) identified 691 potential hepatokines in PHH. Subsequently, pathway analysis assigned these proteins to acute phase response, coagulation, and complement system pathways. The secretome of PHH was compared to the secreted proteins of the liver hepatoma cell line HepG2. Although the secretome of PHH and HepG2 cells show a high overlap, the HepG2 secretome rather mirrors the fetal liver with some cancer characteristics. Collectively, our study represents one of the most comprehensive secretome profiling approaches for PHH, allowing new insights into the composition of the secretome derived from primary human material, and points out strength and weakness of using HepG2 cell secretome as a model for the analysis of the human liver secretome.

## 1. Introduction

The liver is the central organ for carbohydrate, lipid, and protein metabolism, which are tightly interconnected with each other [1]. Protein synthesis has a major impact on hepatic glucose homeostasis [2]. In addition to producing intracellular proteins, hepatocytes are also responsible for generating and secreting most of the plasma proteins [1]. The hepatic secretome defines all proteins, which are produced and secreted by the liver. To study the metabolic functions of the human liver, several in vitro approaches have been established. Forty years ago, liver cells were isolated from patients with hepatic tumors, and these tumor cell lines like HepG2 and Hep3B served as exclusive models, which were easy to sustain and study [3,4]. Although tumor cell lines reflect some aspects of the adult human liver [5], they do not mirror numerous liver functions. HepG2 cells do not express the transcripts of many cytochrome P450 subunits (like CYP2C9, CYP2E1, and CYP3A4) and other enzymes (like bile salt export pump and organic anion transporter), which are involved in drug and bile acid metabolism, respectively [6]. Furthermore, HepG2 cells show altered lipid metabolism, and they carry the homozygous variant rs738409 of patatin-like phospholipase domain-containing protein 3 (PNPLA3) gene [7], the variant which is strongly associated with hepatic steatosis [8]. These results encouraged the development of new model systems, which reflect better the metabolic functions of the human liver. Isolated primary human hepatocytes (PHH) [9] are considered as the gold standard for in vitro hepatocyte function [10] and have many advantages compared to tumor cell lines. PHH express relevant hepatic enzymes [6,11] and, upon stimulus, these cells can accumulate and secrete triacylglycerols [7,12]. On the other hand, the rare occasion of liver surgeries from eligible patients and the possible dedifferentiation of PHH during long term culture conditions [13] limit the routine application of PHH. Recent research data emphasized the endocrine function of the liver since it releases specific proteins called hepatokines [14]. Similar to adipokines and myokines [15,16,17], these hepatokines are attributed to play a central role within the crosstalk between organs [18]. Modulations of these complex hepatokine signatures are hypothesized to participate in the development of metabolic diseases [14], which requires a detailed analysis of the hepatokine containing secretome (hepatokinome).

Our aims were (i.) to characterize the secretome of PHH by high-resolution mass spectrometry and (ii.) to compare the secretome of PHH with the secreted proteins of HepG2 cells. Although the intracellular protein composition of PHH has been described [10,13,19], according to our knowledge, there is no such comprehensive data on the secreted protein profile originated from PHH. Here, we used unbiased protein profiling by liquid chromatography-tandem mass spectrometry (LC-MS/MS), which has been, previously, successfully applied to identify the secretome profile of primary human adipocytes, including the exosomal fraction [16,20] and skeletal muscle cells [17,20], respectively.

## 2. Materials and Methods

### 2.1. Materials

Chemicals and solutions were purchased from Sigma-Aldrich (Munich, Germany) and Lonza (Cologne, Germany) unless otherwise stated.

### 2.2. Human Liver Samples

Liver samples were obtained from patients who underwent liver surgeries. Indications for the surgery were a hepatic hemangioma, curative resection of hepatic metastases of colorectal malignancies, or hepatocellular carcinoma. Liver samples were taken from normal, non-diseased tissue during surgery. Informed, written consent was obtained from all participants, and the Ethics Committee of the University of Tübingen approved the protocol (368/2012BO2) according to the Declaration of Helsinki.

### 2.3. Cell Culture

Primary human hepatocytes (PHH) were isolated after surgery by a two-step EDTA/collagenase perfusion technique, as described previously, with the following modifications. To minimize proteolytic enzyme activities during the isolation, the collagenase solution was mixed 1 to 1 with the perfusion solution II [21]. To stop the collagenase digestion, a solution with 20% fetal bovine serum (FBS) in phosphate-buffered saline (PBS) was used. PHH were seeded at 500,000 cells/wells density on rat-tail collagen-coated 12-well plates and cultivated in Williams Medium E (Pan-Biotech, Aidenbach, Germany) containing 10% FBS (Biochrom/Merck, Berlin, Germany), 11 mM glucose (Thermo Fisher Scientific, Darmstadt, Germany), 2 mM glutamine, 100 U/mL penicillin, 100 µg/mL streptomycin, 1 mM pyruvate, 1% non-essential amino acids, 0.8 µg/mL hydrocortisone (Pfizer, Berlin, Germany), and 50 µU/mL insulin (Sigma-Aldrich) [22]. HepG2 cells were cultivated in RPMI-1640 media with 10% FBS, as published previously [23]. Since the presence of FBS could interfere with the proteomics measurements, fresh media without FBS was given to the cells, and 20 h later, the condition media (supernatant) was collected. The conditioned media were centrifuged for five minutes at 800 g at 4 °C to discard cell debris. The supernatants were frozen at −80 °C. Four independent samples were collected from PHH and HepG2 cells, respectively.

### 2.4. Liquid Chromatography Coupled to Tandem Mass Spectrometry (LC-MS/MS)

Proteins of cell culture supernatant (secretome) were concentrated with 3K Amicon Ultra columns (Merck Millipore, Darmstadt, Germany) [17], and protein concentration was determined using Nanodrop (ThermoFisher Scientific). Ten micrograms of total protein were loaded onto SDS-PAGE (10% polyacrylamide, a separation distance of 0.5 cm), stained with Coomassie blue, and protein bands were cut out and digested in-gel with trypsin [24]. Resulting peptides were separated by reversed-phase liquid chromatography (EASY-Spray C18 column, ES803; ID: 75 µm, 50 cm length; ThermoFisher Scientific) using an Ultimate 3000 system (ThermoFisher Scientific). Elution was performed using a linear gradient 4–34% buffer B (0.1% formic acid, 80% acetonitrile (*v*/*v*)) for 100 min, followed by a 20 min increase to 50% buffer B, a 1 min increase to 90% buffer B, and a 10 min wash with buffer B at a flow rate of 300 nL/min. Tandem mass spectrometry was performed on an Orbitrap Fusion™ Lumos™ Tribrid™ mass spectrometer (ThermoFisher Scientific) utilizing data-dependent (DDA) MS/MS scan method. Each 3 s of the complete run full scan spectra were acquired with AGC Target values 4.0e5, in the 350–1600 (*m*/*z*) scan range with a maximum injection time of 50 ms and a resolution of 120,000. Fragmentation of precursor ions, with an intensity threshold of 2.5e4 and a charge state between 2–7, were performed by higher-energy C-trap dissociation (HCD). Dynamic exclusion was set to 30 s to avoid repeated sequencing of identical peptides.

### 2.5. Analysis of LC-MS/MS Data

MS raw data were processed using MaxQuant 1.6.5.0 [25] with the standard contaminant list of the software. Reviewed human (Taxonomy ID 9606, 20,417 proteins) and bovine (Taxonomy ID 9913, 6006 proteins) FASTA files (downloaded on the 8 April 2019) were used as search databases. Peptide intensities were combined on protein level as majority protein identifiers, and at least two unique and/or razor peptides were used for protein identification and quantification. False discovery rate (FDR) for protein identification was set as <0.01. For protein identification, “matching among runs” were allowed. Human contaminants and “non-human” proteins were discarded except albumin. Proteins with the biased origin, which could belong to bovine as well as to human, were kept and marked in the results as possible contaminants due to the uncertain origin of these proteins. For quantification, MaxQuant-generated intensity-based absolute quantification (iBAQ) values and relative iBAQ (riBAQ) values were applied [26]. Proteins, which showed at least three iBAQ values out of four replicates in PHH (1647 proteins) or HepG2 samples (1739 proteins), were further analyzed. To assign proteins as putative secretory protein, and thus potential hepatokine, identified protein sequences were further analyzed by SignalP 4.1 (http://www.cbs.dtu.dk/services/SignalP/), predicting a signal peptide (SP+), or SecretomeP 2.0 (http://www.cbs.dtu.dk/services/SecretomeP/), (NNscore cut-off: 0.5) predicting non-classical secretory proteins without signal peptide (SP−) [17,27,28]. Proteins, which did not pass the SP+/SP− algorithms, are marked as “non-secretory” proteins (NP). As human plasma database, Peptide Atlas (http://www.peptideatlas.org/) was applied using human plasma (20190320-024847) non-glyco 2017-04 database. Pathway enrichment analysis was performed with g:Profiler (https://biit.cs.ut.ee/gprofiler), which is a web server for functional enrichment, as it was reported previously [29]. Briefly, the secreted 691 PHH and 745 HepG2 proteins were used separately as input sequences and loaded into g: Profiler. In advanced options, Benjamini-Hochberg FDR correction was used with the significance threshold *p* < 0.05. Selected significant Kyoto encyclopedia of genes and genomes term (KEGG) and biological process gene ontology (GO) terms are shown.

## 3. Results

**Figure 1:**
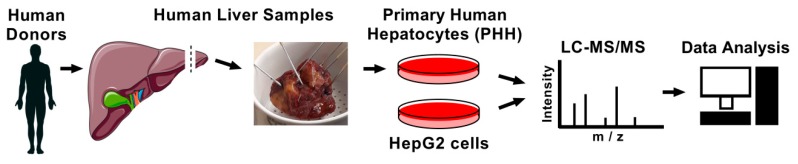
Flow-chart work scheme. Primary human hepatocytes (PHH) were isolated from four human donors. FBS-free condition media were collected from PHH, as well as HepG2 cells, and samples were loaded onto SDS-PAGE, and proteins were in-gel digested. Peptides were separated and measured with liquid chromatography coupled to tandem mass spectrometry. Data were analyzed with MaxQuant.

To characterize the secretome of primary human hepatocytes (PHH), the condition media were collected and subjected to liquid chromatography coupled to tandem mass spectrometry (LC-MS/MS) (Figure 1).

Using LC-MS/MS, 1647 proteins were consistently identified in the condition media of PHH (Table S1). From these proteins, 691 proteins were detected as potentially secreted (with or without signal peptide: SP+ or SP−, respectively) using SignalP and Secretome P algorithms. To determine the proportion of these secreted proteins related to the complete secretome, relative iBAQ (riBAQ) values were calculated (Table S2). Knowledge-based pathway analysis revealed that these putative secreted proteins were involved in acute phase response, coagulation, and complement system (Table 1). Furthermore, pathways for carbohydrate, lipid, and protein metabolism were also significantly enriched among these secreted proteins (Table 1).

**Figure 2:**
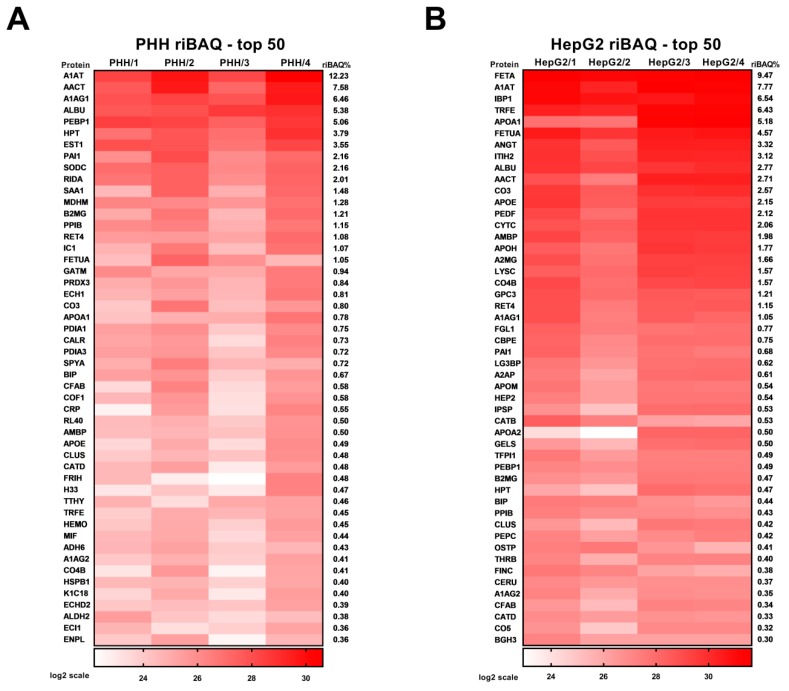
(**A**) PHH (primary human hepatocytes) riBAQ (relative intensity-based absolute quantification) list—top 50 proteins. In PHH samples, 691 secreted proteins were identified and quantified, from which riBAQ could be calculated using MaxQuant for at least three out of four replicates. (**A**) shows the top 50 proteins with the highest riBAQ values, average values of the replicates and protein names are shown in Table 2. (**B**) HepG2 riBAQ list—top 50 proteins. In HepG2 samples, 745 secreted proteins were identified and quantified, from which riBAQ could be calculated using MaxQuant for at least three out of four replicates. (**B**) shows the top 50 proteins with the highest riBAQ values, average values of the replicates and protein names are shown in Table 3. Heat maps show log_2_ data.

The first 50 PHH secreted proteins with the highest riBAQ abundance are shown in Table 2 and Figure 2A. These 50 highest abundant proteins represent 76.9% of total PHH secreted proteins.

In the secretome of HepG2 cells, 1739 proteins were consistently identified (Table S1). SignalP and Secretome P algorithms detected 745 potentially secreted proteins, for which riBAQ values were calculated. The first 50 proteins with the highest riBAQ abundance are shown in Table 3 and Figure 2B, whereas the complete data set for HepG2 cells is depicted in Table S3. These 50 highest abundant proteins represent 86.1% of total HepG2 secreted proteins. Specific proteins for PHH and HepG2 cells are shown in Table S4.

From the both top 50 secreted PHH and top 50 secreted HepG2 proteins, 48 proteins (96%) have been already identified in human plasma (Peptide Atlas). Comparing the top 50 secreted proteins of PHH and HepG2 cells, 22 proteins (marked in bold in Table 2 and Table 3) were common. From the top 50 PHH list, 48 proteins (96%) were found among the 745 HepG2 proteins, whereas from the top 50 HepG2 list, 48 proteins (96%) were found among the 691 PHH proteins. C-reactive protein and Serum amyloid A-1 protein were only found in PHH samples, whereas Glypican-3 and Gastricsin were only detected in HepG2 cells (marked underlined in Table 2 and Table 3). These results suggest that the secretome of HepG2 cells mainly resembles the secreted protein profile of PHH; however, major differences also exist between them.

To compare the function of the PHH and HepG2 secreted proteins, KEGG pathway analysis was performed. Analyzing the 691 PHH secreted and 745 HepG2 secreted proteins separately, we observed that several liver pathways were significantly enriched in both data sets like cholesterol metabolism, complement and coagulation cascades, and amino acid pathways (Table 4, upper table). On the other hand, glycosphingolipid biosynthesis was only enriched in HepG2 secretome, whereas specific amino acid pathways (arginine, proline, and alanine metabolism), as well as fatty acid degradation pathway, were only enriched in the secretome of PHH (Table 4, upper table). To study whether these pathways are also reflected among the proteins, which are specific for PHH (64 proteins) or HepG2 cells (101 proteins) (Table S4), new KEGG pathway analyses were accomplished with these PHH and HepG2 specific data sets separately. These analyses confirmed the former results that the specific PHH secreted proteins were significantly enriched in amino acid and ketone bodies pathways, which were not enriched in the specific secreted proteins of HepG2 cells (Table 4, lower table). The glycosphingolipid biosynthesis pathway was, however, significantly enriched in HepG2 secretome, which was not enriched among the secreted PHH proteins (Table 4, lower table). These data suggest that although the secretome of PHH and HepG2 cells show some similarities, they are not identical. Compared to HepG2 cells, PHH is probably better equipped for amino acid and fatty acid metabolism.

## 4. Discussion

In the liver, protein and glucose metabolism are intimately connected since amino acids serve as precursors for gluconeogenesis [2]. Furthermore, the liver secretes key molecules, the hepatokines, which regulate lipid and glucose homeostasis in the liver and also in skeletal muscle and other tissues [30]. Therefore, it is inevitable to determine the secreted proteins (secretome), which are produced by primary human hepatocytes. In contrast to the intracellular milieu, the measurement of the secretome is limited due to the low signal to noise ratio, making the detection of low-abundance proteins difficult against the highly abundant serum-containing proteins [31,32]. To overcome these challenges, we applied our well-established mass spectrometry-based profiling approach for tissue-specific secretomes [16,17,20]. Utilizing label-free LC-MS/MS and bioinformatics filter methods, from 1647 proteins, 691 were identified in the secretome of PHH as putative secreted liver proteins. To enable reliable comparison and calculation of relative protein intensities (iBAQ values), in the PHH iBAQ protein list, only those proteins were included, for which the iBAQ values could be calculated for at least three out of four biological replicates. In our study, we focused on proteins exhibiting a signal peptide (SP+) or showing sequence features for non-classical secretion (SP−). Nevertheless, there is a growing body of evidence that proteins frequently assigned as “non-secretory” (NP) play an important role in the global cellular secretome [16] and do not necessarily represent contaminations. It was shown that the complex intercellular communication is also mediated by extracellular vesicles, responsible for the unconventional secretion of proteins [33,34].

Using 2D-PAGE and shotgun proteomics, Slany and colleagues previously identified 72 proteins in the condition media of PHH [35]. From these proteins, 36 were found in the 691 possibly secreted PHH protein list representing 5% of 691 proteins. Among the secreted PHH proteins in our study, we found several previously described hepatokines, which showed metabolic functions in rodents and cell lines [30]. These hepatokines are Fetuin-A, Sex hormone-binding globulin, Angiopoietin-related protein 4, Retinol-binding protein 4, and Selenoprotein P, which, however, were not identified in the condition media of PHH in the study of Slany et al. except Fetuin-A [35]. In a recent study, the secretome of the human liver hepatocolangiocarcinoma HepaRG cell line, which was isolated from a patient with hepatitis C infection, was described [36]. One should note that although HepaRG cells express many drug-metabolizing enzymes [37], they show abnormal karyotypic alterations, probably due to the cancer characteristics [6]. In the secretome of HepaRG cells, 313 proteins were identified, and four out of the five previously mentioned hepatokines were also present among them [36]. From the 691 PHH secreted proteins identified in our study, 163 proteins were detected in the secretome of HepaRG, which indicates that 76% of the PHH secreted proteins have not been described yet.

In addition to the secreted PHH proteins, we also compared the PHH secretome with the secreted protein profile of hepatoma cell line HepG2. Since the majority of proteins were common in both PHH and HepG2 secretome, these data indicate that the secreted proteome show many similarities between PHH and HepG2 cells, as it was previously shown for the intracellular proteome [10]. This was also confirmed by pathway analysis (Table 4, upper panel), which showed that lipid metabolism, complement and coagulation pathways, and valine, leucine, and isoleucine amino acid metabolic pathways were enriched among the secreted proteins of PHH and HepG2 cells. On the other hand, some fatty acid and amino acid metabolism pathways were only enriched in the secretome of PHH (Table 4, upper panel).

As an indirect comparison of PHH and HepG2 secretome, we further analyzed specific proteins, which were only detected in PHH or HepG2 supernatants. Our results revealed 64 PHH and 101 HepG2 specific proteins (Table S4). KEGG pathway analysis of these specific proteins demonstrated that the secreted proteins of PHH showed enrichment in specific fatty acid and amino acid pathways, which was not detected in HepG2 samples (Table 4, lower panel). These results suggest that PHH are probably better equipped for amino acid and fatty acid metabolism. Our data also showed that the acute phase proteins, C-reactive protein (CRP) and Serum amyloid A-1 protein (SAA1), belong to the top 50 PHH secreted proteins; however, they were lacking in the secretome of HepG2 cells (Table S1). The lack of CRP production in HepG2 cells may be explained by the more immature hepatic status of HepG2 cells, which is in agreement with the observation that preterm newborns compared to term newborns show lower CRP response [38]. The protein SAA1 was reported in the intracellular proteome of PHH but was not detected in HepG2 cells [36]. These results indicate that HepG2 cells show altered expression or secretion of acute-phase proteins. We also found that from the top 50 HepG2 secreted proteins, Gastricsin and Glypican-3 were not detected in the secretome of PHH (Table S1). Gastricsin was recently postulated to play a tumorigenesis role in the progression of hepatocellular carcinoma (HCC) [39]. Glypican-3 is among the most promising candidates for early diagnostic markers of HCC [40]. Furthermore, two other proteins—Midkine and Vascular endothelial growth factor A—which were implicated in the development of HCC, were also exclusively expressed in HepG2 secretome (Table S1). All these four proteins were also observed to be specific for the secreted proteins of HepG2 cells and missing in the secretome of PHH by a previous study [35] validating our data. Among the HepG2 specific secreted proteins, the glycosphingolipid biosynthesis pathway was significantly enriched (Table 4, lower panel). Glycosphingolipid metabolism was shown to be altered in many cancers, and glycosphingolipid synthesis inhibition is considered as a potential therapeutic target for HCC [41]. These results suggest that the HepG2 secretome shows characteristics for cancer metabolism, which was not found in the secretome of PHH. Furthermore, Rowe and colleagues investigated the intracellular proteome of human primary fetal and adult hepatocytes, as well as HepG2 cells, and the authors found that many protein changes of HepG2 cells compared with adult hepatocytes were reflected by parallel alterations in the comparison of fetal hepatocytes—adult hepatocytes [10]. These results indicate that HepG2 cells, in terms of protein composition, rather resemble fetal than adult hepatocytes.

Some of the former proteomics studies performed a quantitative comparison of intracellular proteins between PHH and HepG2 cells [10,19,36]. In our study, we applied label-free quantification and calculated iBAQ values, which are used for the estimation of protein content in relation to total proteins in one sample, but they are not routinely applied for the quantification of one protein between two biological samples [26]. Therefore, we generated two independent lists for the PHH and HepG2 cells secreted proteins and did not compare iBAQ values directly between PHH and HepG2 samples. If we build iBAQ ratios for the individual proteins as PHH average iBAQ/HepG2 average iBAQ, then some previously described quantitative differences in the intracellular protein composition between PHH and HepG2 cells could be reflected by the iBAQ ratios of secreted proteins. For example, the intracellular protein levels for Liver carboxylesterase 1, 2-iminobutanoate/2-iminopropanoate deaminase and Alcohol dehydrogenase 6 were higher in PHH compared to HepG2 cells [10,19], which were also observed in the secretomes of our study as positive PHH average iBAQ/HepG2 average iBAQ ratios (Table S1). These results may suggest that some aspects of the quantitative intracellular differences between PHH and HepG2 cells could be reflected by the secretome.

Altogether our data first provide a comprehensive description of the secretome derived from PHH, which mirrors many metabolic processes, relevant for the adult liver function. The provided protein catalog allows new insights into the complexity of the hepatokinome and paves the way to select new targets for further analysis of inter-organ communication. Secondly, the comparison of PHH and HepG2 cells secretome shows a high overlap. Nevertheless, in detail, the secretome of HepG2 cells rather reflects the fetal liver with special cancer characteristics, which has to be considered, when HepG2 cells are used as a model for secretome studies.

## Figures and Tables

**Table 1:** Pathway enrichment of PHH secretome.

Term ID	Term Name	adj. *p*-Value
GO:0006956	complement activation	<0.0001
GO:0006958	complement activation, classical pathway	<0.0001
GO:0006957	complement activation, alternative pathway	<0.0001
GO:0007596	blood coagulation	<0.0001
GO:0007597	blood coagulation, intrinsic pathway	<0.0001
GO:0072378	blood coagulation, fibrin clot formation	<0.0001
GO:0042730	fibrinolysis	<0.0001
GO:0006953	acute-phase response	<0.0001
GO:0002526	acute inflammatory response	<0.0001
GO:0019538	protein metabolic process	<0.0001
GO:0051246	regulation of protein metabolic process	<0.0001
GO:1901605	alpha-amino acid metabolic process	<0.0001
GO:1901135	carbohydrate derivative metabolic process	<0.0001
GO:0006629	lipid metabolic process	<0.0001
GO:0097006	regulation of plasma lipoprotein particle levels	<0.0001
GO:0034369	plasma lipoprotein particle remodeling	<0.0001

The 691 secreted proteins identified in the primary human hepatocytes (PHH) secretome were subjected to pathway analysis using g: Profiler, selected pathways are shown. Term identification numbers (Term ID) represent biological process gene ontology (GO) terms. adj. *p*-value: Benjamini-Hochberg adjusted *p*-value.

**Table 2:** PHH riBAQ list—top 50 proteins.

Prot. ID	Prot. Name	Prot. Symb.	Possib. Contam. ^1^	av iBAQ ^2^	riBAQ (%) ^3^
**P01009**	**Alpha-1-antitrypsin**	**A1AT**		**789277500**	**12.23**
**P01011**	**Alpha-1-antichymotrypsin**	**AACT**		**489095000**	**7.58**
**P02763**	**Alpha-1-acid glycoprotein 1**	**A1AG1**		**417130000**	**6.46**
**P02768**	**Serum albumin**	**ALBU**		**346990000**	**5.38**
**P30086**	**Phosphatidylethanolamine-binding protein 1**	**PEBP1**		**326665000**	**5.06**
**P00738**	**Haptoglobin**	**HPT**		**244395000**	**3.79**
P23141	Liver carboxylesterase 1	EST1		229112500	3.55
**P05121**	**Plasminogen activator inhibitor 1**	**PAI1**		**139604500**	**2.16**
P00441	Superoxide dismutase [Cu-Zn]	SODC		139590750	2.16
P52758	2-iminobutanoate/2-iminopropanoate deaminase	RIDA		129657750	2.01
P0DJI8	Serum amyloid A-1 protein	SAA1		95757500	1.48
P40926	Malate dehydrogenase, mito.	MDHM	x	82348750	1.28
**P61769**	**Beta-2-microglobulin**	**B2MG**		**78243000**	**1.21**
**P23284**	**Peptidyl-prolyl cis-trans isomerase B**	**PPIB**		**73998500**	**1.15**
**P02753**	**Retinol-binding protein 4**	**RET4**		**69459500**	**1.08**
P05155	Plasma protease C1 inhibitor	IC1		68893500	1.07
**P02765**	**Fetuin-A**	**FETUA**		**67909500**	**1.05**
P50440	Glycine amidinotransferase, mito.	GATM		60424000	0.94
P30048	Thioredoxin-dependent peroxide reductase, mito.	PRDX3		53912250	0.84
Q13011	Delta(3,5)-Delta(2,4)-dienoyl-CoA isomerase, mito.	ECH1		52252250	0.81
**P01024**	**Complement C3**	**CO3**		**51702000**	**0.80**
**P02647**	**Apolipoprotein A-I**	**APOA1**		**50100750**	**0.78**
P07237	Protein disulfide-isomerase	PDIA1	x	48349000	0.75
P27797	Calreticulin	CALR		47263000	0.73
P30101	Protein disulfide-isomerase A3	PDIA3		46786500	0.72
P21549	Serine-pyruvate aminotransferase	SPYA		46203500	0.72
**P11021**	**Endoplasmic reticulum chaperone BiP**	**BIP**	**x**	**43119250**	**0.67**
**P00751**	**Complement factor B**	**CFAB**		**37699500**	**0.58**
P23528	Cofilin-1	COF1	x	37466000	0.58
P02741	C-reactive protein	CRP		35494325	0.55
P62987	Ubiquitin-60S ribosomal protein L40	RL40	x	32257250	0.50
**P02760**	**Protein AMBP**	**AMBP**		**32188750**	**0.50**
**P02649**	**Apolipoprotein E**	**APOE**		**31445250**	**0.49**
**P10909**	**Clusterin**	**CLUS**		**31080000**	**0.48**
**P07339**	**Cathepsin D**	**CATD**		**30845450**	**0.48**
P02794	Ferritin heavy chain	FRIH		30716925	0.48
P84243	Histone H3.3	H33	x	30172300	0.47
P02766	Transthyretin	TTHY		29464250	0.46
**P02787**	**Serotransferrin**	**TRFE**		**28945750**	**0.45**
P02790	Hemopexin	HEMO		28893000	0.45
P14174	Macrophage migration inhibitory factor	MIF		28618500	0.44
P28332	Alcohol dehydrogenase 6	ADH6		27676000	0.43
**P19652**	**Alpha-1-acid glycoprotein 2**	**A1AG2**		**26294000**	**0.41**
**P0C0L5**	**Complement C4-B**	**CO4B**		**26207125**	**0.41**
P04792	Heat shock protein beta-1	HSPB1		25729750	0.40
P05783	Keratin, type I cytoskeletal 18	K1C18		25491850	0.40
Q86YB7	Enoyl-CoA hydratase domain-containing protein 2, mito.	ECHD2	x	25239750	0.39
P05091	Aldehyde dehydrogenase, mito.	ALDH2		24239250	0.38
P42126	Enoyl-CoA delta isomerase 1, mito.	ECI1		23068000	0.36
P14625	Endoplasmin	ENPL	x	23033875	0.36

In primary human hepatocyte (PHH) samples, 691 secreted proteins were identified and quantified, from which relative intensity-based absolute quantification (riBAQ) could be calculated using MaxQuant for at least three out of four biological replicates. Table 2 shows the top 50 proteins with the highest average of riBAQ values, and single values of the replicates are shown in Figure 2A. Data for the other proteins are shown in Table S2. Protein names in bold depict proteins, which were also identified and quantified among the top 50 proteins in the secretome of HepG2 cells. Protein names underlined depict PHH specific proteins, which were not identified in HepG2 samples. **^1^** In possible contamination (Possib. Contam.) column, “x” denotes proteins, which could probably belong to bovine as well as to human species. **^2^** The average of iBAQ values (av iBAQ) was calculated from three-four replicates. **^3^** riBAQ values were calculated by dividing the average iBAQ values of the appropriate proteins with the sum of iBAQ values of all quantified proteins and shown as relative proportion in %. Prot.: Protein, Symb.: symbol, mito.: mitochondrial.

**Table 3:** HepG2 riBAQ list—top 50 proteins.

Prot. ID	Prot. Name	Prot. Symb.	Possib. Contam. ^1^	av iBAQ ^2^	riBAQ (%) ^3^
P02771	Alpha-fetoprotein	FETA		2998500000	9.47
**P01009**	**Alpha-1-antitrypsin**	**A1AT**		**2460775000**	**7.77**
P08833	Insulin-like growth factor-binding protein 1	IBP1		2070400000	6.54
**P02787**	**Serotransferrin**	**TRFE**		**2035550000**	**6.43**
**P02647**	**Apolipoprotein A-I**	**APOA1**		**1639730000**	**5.18**
**P02765**	**Fetuin-A**	**FETUA**		**1446420000**	**4.57**
P01019	Angiotensinogen	ANGT		1050527500	3.32
P19823	Inter-alpha-trypsin inhibitor heavy chain H2	ITIH2		986895000	3.12
**P02768**	**Serum albumin**	**ALBU**		**876332500**	**2.77**
**P01011**	**Alpha-1-antichymotrypsin**	**AACT**		**859190000**	**2.71**
**P01024**	**Complement C3**	**CO3**		**813957500**	**2.57**
**P02649**	**Apolipoprotein E**	**APOE**		**679975000**	**2.15**
P36955	Pigment epithelium-derived factor	PEDF		672652500	2.12
P01034	Cystatin-C	CYTC		653532500	2.06
**P02760**	**Protein AMBP**	**AMBP**		**627792500**	**1.98**
P02749	Beta-2-glycoprotein 1	APOH		560445000	1.77
P01023	Alpha-2-macroglobulin	A2MG		526075000	1.66
P61626	Lysozyme C	LYSC		498212500	1.57
**P0C0L5**	**Complement C4-B**	**CO4B**		**496745000**	**1.57**
P51654	Glypican-3	GPC3		382685000	1.21
**P02753**	**Retinol-binding protein 4**	**RET4**		**363022500**	**1.15**
**P02763**	**Alpha-1-acid glycoprotein 1**	**A1AG1**		**331710000**	**1.05**
Q08830	Fibrinogen-like protein 1	FGL1		243105000	0.77
P16870	Carboxypeptidase E	CBPE		236250000	0.75
**P05121**	**Plasminogen activator inhibitor 1**	**PAI1**		**214605000**	**0.68**
Q08380	Galectin-3-binding protein	LG3BP		195722250	0.62
P08697	Alpha-2-antiplasmin	A2AP		193182750	0.61
O95445	Apolipoprotein M	APOM		170078750	0.54
P05546	Heparin cofactor 2	HEP2		169633750	0.54
P05154	Plasma serine protease inhibitor	IPSP		167712250	0.53
P07858	Cathepsin B	CATB		167710250	0.53
P02652	Apolipoprotein A-II	APOA2		159891350	0.50
P06396	Gelsolin	GELS		157569250	0.50
P10646	Tissue factor pathway inhibitor	TFPI1		156584750	0.49
**P30086**	**Phosphatidylethanolamine-binding protein 1**	**PEBP1**		**153822500**	**0.49**
**P61769**	**Beta-2-microglobulin**	**B2MG**		**150395750**	**0.47**
**P00738**	**Haptoglobin**	**HPT**		**147916750**	**0.47**
**P11021**	**Endoplasmic reticulum chaperone BiP**	**BIP**	**x**	**139597250**	**0.44**
**P23284**	**Peptidyl-prolyl cis-trans isomerase B**	**PPIB**		**134877500**	**0.43**
**P10909**	**Clusterin**	**CLUS**		**134223250**	**0.42**
P20142	Gastricsin	PEPC		131868250	0.42
P10451	Osteopontin	OSTP		129970500	0.41
P00734	Prothrombin	THRB		127123250	0.40
P02751	Fibronectin	FINC		120668000	0.38
P00450	Ceruloplasmin	CERU		117001250	0.37
**P19652**	**Alpha-1-acid glycoprotein 2**	**A1AG2**		**111402750**	**0.35**
**P00751**	**Complement factor B**	**CFAB**		**108606250**	**0.34**
**P07339**	**Cathepsin D**	**CATD**		**104733250**	**0.33**
P01031	Complement C5	CO5		100732500	0.32
Q15582	Transforming growth factor-beta-induced protein ig-h3	BGH3		95466000	0.30

In HepG2 samples, 745 secreted proteins were identified and quantified, from which relative intensity-based absolute quantification (riBAQ) could be calculated using MaxQuant for at least three out of four replicates. Table 3 shows the top 50 proteins with the highest riBAQ values, and single values of the replicates are shown in Figure 2B. Data for the other proteins are shown in Table S3. Protein names in bold depict proteins, which were also identified and quantified among the top 50 proteins in the secretome of PHH. Protein names underlined depict HepG2 specific proteins, which were not identified in PHH samples. **^1^** In possible contamination (Possib. Contam.) column, “x” denotes proteins, which could probably belong to bovine as well as to human species. **^2^** The average of iBAQ values (av iBAQ) was calculated from three-four replicates. **^3^** riBAQ values were calculated by dividing the average iBAQ values of the appropriate proteins with the sum of iBAQ values of all quantified proteins and shown as relative proportion in %. Prot.: Protein, Symb.: symbol.

**Table 4:** Pathway enrichment of PHH and HepG2 secretomes.

		**PHH 691 prot.**	**HepG2 745 prot.**
**Term ID**	**Term Name**	**adj. *p*-value**	**adj. *p*-value**
KEGG:04979	Cholesterol metabolism	<0.0001	<0.0001
KEGG:04610	Complement and coagulation cascades	<0.0001	<0.0001
KEGG:04141	Protein processing in endoplasmic reticulum	<0.0001	<0.0001
KEGG:00280	Valine, leucine and isoleucine degradation	<0.0001	0.0020
KEGG:00603	*Glycosphingolipid biosynthesis-globo and isoglobo series*	-	0.0216
KEGG:00520	*Amino sugar and nucleotide sugar metabolism*	0.0176	-
KEGG:00330	*Arginine and proline metabolism*	0.0185	-
KEGG:00410	*Beta-alanine metabolism*	0.0183	-
KEGG:00071	*Fatty acid degradation*	0.0107	-
		**PHH 64 prot.**	**HepG2 101 prot.**
**Term ID**	**Term Name**	**adj. *p*-value**	**adj. *p*-value**
KEGG:00472	*D-Arginine and D-ornithine metabolism*	0.0489	-
KEGG:00340	*Histidine metabolism*	0.0489	-
KEGG:00072	*Synthesis and degradation of ketone bodies*	0.0202	-
KEGG:00601	*Glycosphingolipid biosynthesis-lacto and neolacto series*	-	0.0010

In the upper table, 691 secreted proteins from the primary human hepatocytes (PHH) iBAQ list and 745 secreted proteins from the HepG2 iBAQ list were used as input proteins. In the lower table, 64 PHH specific proteins (part of 691 PHH iBAQ list) and 101 HepG2 specific proteins (part of 745 HepG2 iBAQ list) were used as input proteins. Pathway analysis was performed for the indicated input proteins separately using g: Profiler, and selected pathways are shown. Pathways in italics depict specific pathways. prot.: protein, adj. *p*-value: Benjamini-Hochberg adjusted *p*-value. KEGG: Kyoto encyclopedia of genes and genomes term.

## Supplementary Materials

The following data are available online at https://www.mdpi.com/2072-6643/11/8/1795/s1, Table S1: Proteomics data, Table S2: PHH riBAQ list, Table S3: HepG2 riBAQ list, Table S4: PHH and HepG2 specific proteins.
